# Nitrosative Stress Molecules in Multiple Sclerosis: A Meta-Analysis

**DOI:** 10.3390/biomedicines9121899

**Published:** 2021-12-14

**Authors:** Moritz Förster, Christopher Nelke, Saskia Räuber, Hans Lassmann, Tobias Ruck, Maria Pia Sormani, Alessio Signori, Hans-Peter Hartung, Patrick Küry, Sven G. Meuth, David Kremer

**Affiliations:** 1 Department of Neurology, Medical Faculty, Heinrich Heine University, 40225 Düsseldorf, Germany; moritz.foerster@med.uni-duesseldorf.de (M.F.); christopherjannik.nelke@med.uni-duesseldorf.de (C.N.); saskiajanina.raeuber@med.uni-duesseldorf.de (S.R.); Tobias.Ruck@med.uni-duesseldorf.de (T.R.); Hans-Peter.Hartung@med.uni-duesseldorf.de (H.-P.H.); kuery@uni-duesseldorf.de (P.K.); SvenGuenther.Meuth@med.uni-duesseldorf.de (S.G.M.); 2 Department of Neuroimmunology, Center for Brain Research, Medical University of Vienna, 1090 Vienna, Austria; hans.lassmann@meduniwien.ac.at; 3 Department of Health Sciences, University of Genoa, 16121 Genoa, Italy; mariapia.sormani@unige.it (M.P.S.); alessio.signori.unige@gmail.com (A.S.); 4 IRCCS Ospedale Policlinico San Martino, 16121 Genoa, Italy; 5 Brain and Mind Center, University of Sydney, Sydney 2006, Australia; 6 Department of Neurology, Medical University of Vienna, 1090 Vienna, Austria; 7 Department of Neurology, Palacky University Olomouc, 77900 Olomouc, Czech Republic

**Keywords:** multiple sclerosis, biomarker, nitrosative stress, NOx, meta-analysis

## Abstract

Multiple sclerosis (MS) is an immune-mediated disease of the central nervous system of unknown etiology. As it is still a diagnosis of exclusion, there is an urgent need for biomarkers supporting its diagnosis. Increasing evidence suggests that nitrosative stress may play a pivotal role in the pathogenesis of MS. However, previous reports supporting the role of nitrosative stress molecules as disease biomarkers are inconsistent overall. We therefore systematically analyzed the existing literature to compare the serum and cerebrospinal fluid (CSF) levels of nitrite/nitrate in MS patients with those in patients with noninflammatory other neurological diseases (NIOND) and healthy controls (HC), respectively. We searched the PubMed database and included original articles investigating nitrite/nitrate levels in MS patients and NIOND patients or HC based on predefined selection criteria. Effect sizes were estimated by the standardized mean difference using a random effects model. Our results suggest that MS is associated with higher nitrite/nitrate levels within the CSF compared with patients with NIOND (SMD of 1.51; 95% CI: 0.72, 2.30; *p* = 0.0008). Likewise, nitrite/nitrate in the CSF of MS patients trends towards increased levels compared with those of HC but does not reach statistical significance (SMD of 3.35; 95% CI: −0.48, 7.19; *p* = 0.07). Measurement of nitrite/nitrate in the CSF might be a valuable tool facilitating the differentiation of MS and NIOND. Further studies with more homogeneous study criteria are needed to corroborate this hypothesis.

## 1. Introduction

Multiple sclerosis (MS) is an immune-mediated inflammatory disease of the central nervous system (CNS) of unclear etiology. It is characterized by inflammatory, demyelinating, and degenerative aspects and can be divided into different subtypes, such as a relapsing-remitting (RRMS), a primary progressive (PPMS), and a secondary progressive (SPMS) form. Pathomechanistically, repeated inflammatory (auto)immune attacks on the CNS lead to oligodendrocyte and myelin sheath damage, resulting in impaired axonal signal conduction. Inflammation is mediated by reactive immune cells comprising not only T lymphocytes and B cells but also CNS-resident activated microglia and macrophages [1,2,3]. Accumulating evidence suggests that oxidative and nitrosative stress molecules produced by immune or CNS-resident cells play a pivotal role in the pathogenesis and progression of MS. These molecules include oxygen and nitrogen radicals, such as superoxide anions (O2^−^), hydroxyl radicals (OH^−^), or nitric oxide (NO). NO contributes to vasodilation and disruption of the blood–brain barrier (BBB), which is one of the core elements of MS histopathology [4]. It facilitates the transmigration of leukocytes into the CNS and fuels continuing inflammation. NO is a volatile gaseous molecule that is only mildly damaging. However, by reacting with O2^−^, it forms the extremely toxic derivate peroxynitrite (ONOO^−^). Via its oxidative and nitrosative properties, ONOO^−^ damages numerous molecules, such as DNA and lipids, rendering them inoperable [5,6]. Thus, NO is key for demyelination and axonal degeneration by indirectly impairing oligodendroglial energy metabolism via mitochondrial DNA and lipid membrane damage. This ultimately results in oligodendroglial cell death and myelin demise. In addition, NO-mediated protein nitration can activate or inactivate proteins or even cause them to gain new functions [7]. For instance, NO-derived species can activate matrix metalloproteinases (MMPs), which degrade myelin components [8]. Moreover, they can impair the metabolic support of axons through downregulation of the oligodendroglial monocarboxylate transporter 1 (MCT1) [9] or lead to an accumulation of nitrosylated protein residues in MS lesions, such as 3-nitrotyrosine [10,11]. NO is quantifiable in its soluble forms of nitrate or nitrite, which are stored in biofluids, such as the cerebrospinal fluid (CSF) or the serum. This is relevant for the clinician as the CSF is one of the pillars of MS diagnosis and has regained substantial importance since the revision of the McDonald diagnostic criteria in 2017 [12]. In this context, it is important for the clinician to rule out MS mimics, such as noninflammatory other neurological diseases (NIOND) like migraine or small vessel disease. Similar to MS, in these diseases, patients complain of relapsing symptoms and feature white matter lesions on cerebral MR imaging. At times, the unequivocal classification of such lesions can constitute a challenge. Moreover, beyond oligoclonal bands (OCBs) there are no established CSF markers that support the diagnosis of MS, even though the past years have seen the MRZ reaction gain a place as a highly specific diagnostic tool. Therefore, an additional biomarker supporting the diagnosis of MS would be valuable regarding an earlier diagnosis and thus a more efficient treatment. In this regard, several meta-analyses have investigated the potential of various molecules [13,14,15]. However, the potential of nitrosative stress molecules as disease biomarkers has not yet been systematically investigated. With this meta-analysis, we aim to suggest the measurement of nitrogen species as a tool to better delineate MS from other diseases and healthy conditions.

## 2. Materials and Methods

### 2.1. Search Strategy

This meta-analysis was performed in accordance with the preferred reporting items for systematic reviews and meta-analyses (PRISMA) statement. Peer-reviewed articles in English from the PubMed database were systematically reviewed. The terms used for the database search were “multiple sclerosis” and “nitrate” or “nitrite” or “nitrosative stress” without restriction to the year of publication. Clinical studies were included if data on the mentioned biomarkers for RRMS and NIOND patients and HC were given. Accordingly, studies were not considered if they included data from in vitro or animal experiments. Primary screening of the titles and abstracts was followed by selection of articles being relevant to this study and ensuing full-text scrutiny. Studies with serum and cerebrospinal fluid data were included. For reasons of clinical practicability, two comparison groups were formed for the meta-analysis: NIOND versus RRMS patients and HC versus RRMS patients. In cases where no differentiation of MS subtypes was made, we included all available data given for pooled “MS patients”. Moreover, in cases where differentiation was made between “active” and “stable” MS, we only included “active” MS. Studies were excluded if (1) no result parameters were given in the text, (2) the study design did not correspond to the envisaged design of a comparison of NIOND versus MS patients or HC versus MS patients and/or the study investigates the effect of a medication, and (3) not nitrites/nitrates but other molecules were investigated, such as NO. To calculate the effective size (ES) for this meta-analysis data of mean biomarker concentration, standard deviation (SD) and sample size were extracted from the included studies. Data on age, sex, disease duration, and expanded disability status scale were extracted as well.

### 2.2. Data Extraction

We extracted data including first author, publication year, study design, number of patients per group, selection of patients and controls, and recorded levels of the nitrosative stress species nitrite and nitrate and the sum of both (NOx).

### 2.3. Data Analysis

Meta-analysis was performed with R 3.5.3 (R Foundation for Statistical Computing, Vienna, Austria) using the metafor package [16]. Heterogeneity was assessed by calculating the *I*^2^ value [17]. An *I*^2^ value greater 50% was considered a high degree of heterogeneity, and a random-effects model was applied [18]. We calculated the pooled effect estimate and 95% confidence interval (CI) for each between group comparisons. Subgroup analysis was performed for individual nitrosative stress molecules. A *p*-value < 0.05 was considered statistically significant.

## 3. Results

### 3.1. Inclusion of Studies

**Figure 1:**
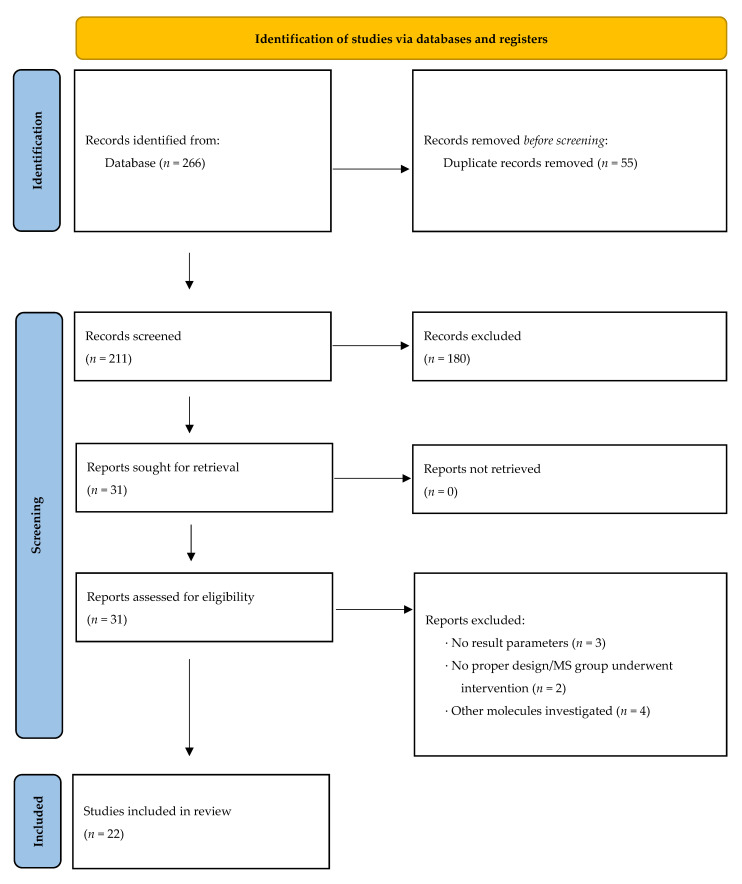
Preferred reporting items for systematic reviews and meta-analyses (PRISMA) study flow diagram.

Based on our search strategy, we identified 266 studies from the PubMed database. After removing duplicates, the number of records eligible for screening was 211. Out of these, 180 did not meet the inclusion criteria; thus, 31 were further assessed for eligibility (see Methods). Nine studies were excluded due to various reasons (see Figure 1 and Methods). The remaining 22 studies were included in the final analysis. The selection process is illustrated in Figure 1 and further characterized in the Methods Section. Regarding CSF analysis in MS and NIOND patients, 3 studies investigated nitrite levels; 4, nitrate levels; and 12, the sum of both. No study analyzed serum nitrite levels in MS patients compared with NIOND, while 1 study analyzed serum nitrate levels. Four studies analyzed NOx in the same collective (see Table 1). Comparing MS patients with healthy controls (HC), only 1 study analyzed the nitrite and nitrate concentration in the CSF, while 3 studies analyzed NOx in the CSF. Six studies analyzed the serum concentration of NOx in the same collective. Studies separately examining nitrite or nitrate were not available (see Table 2).

### 3.2. Study and Patient Characteristics

The included studies were published between 1995 and 2020. All studies used valid diagnostic criteria for MS, such as the McDonald or Poser criteria. The first comparison group examining nitrite, nitrate, or NOx levels in patients with NIOND and MS comprised 348 MS patients and 310 NIOND patients within the CSF subgroup, while in the serum subgroup, 102 MS patients and 83 NIOND patients were included. The second group comparing MS patients and HC included 80 MS patients and 54 HC in the CSF subgroup, while the serum group comprised 373 MS patients and 410 HC. Regarding the analysis technique, 70.8% (17/24) of the studies used Griess reaction, followed by spectrophotometric or semiautomated assays, to measure nitrite and nitrate levels.

### 3.3. Nitrosative Stress Molecules in Patients with MS and NIOND

**Figure 2:**
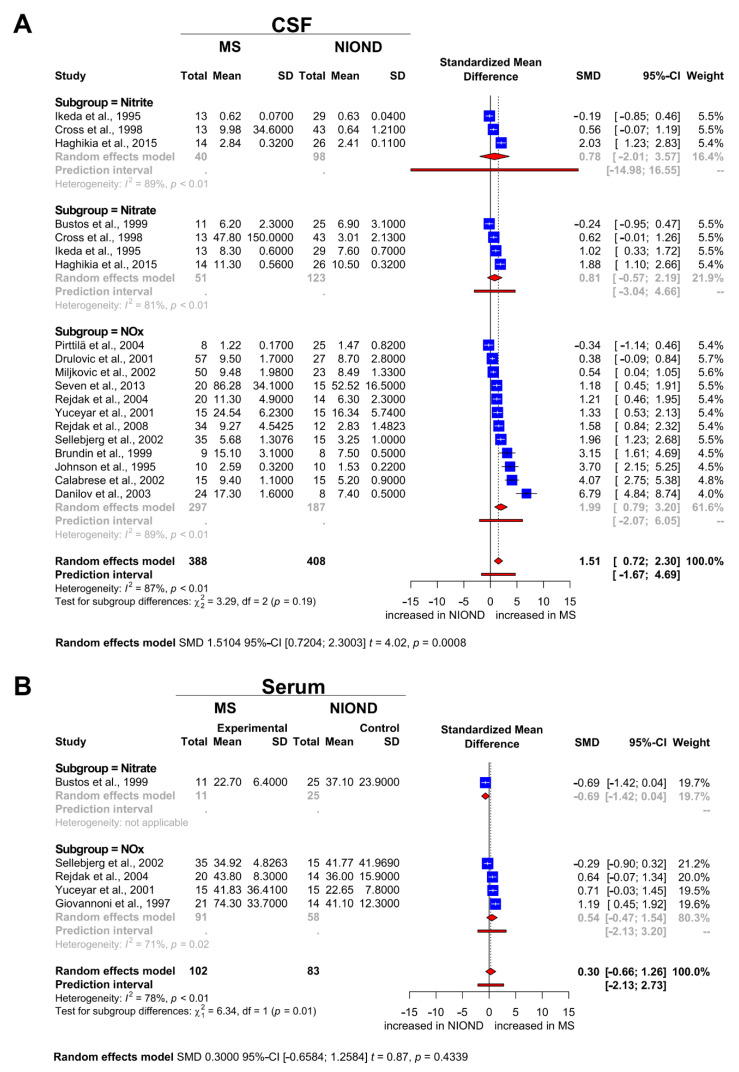
Forest plots displaying the meta-analysis for markers of nitrosative stress in (**A**) the CSF and (**B**) the serum compartment for MS vs. NIOND, respectively. The dashed vertical line indicates the overall effect, while the solid line indicates the null effect (SMD = 0). Abbreviations: CSF = cerebrospinal fluid, NIOND = noninflammatory other neurological disease, NOx = sum of nitrite and nitrate levels, MS = multiple sclerosis, SD = standard deviation, SMD = standardized mean difference.

As elucidated above, for CSF analysis a total of 658 patients (348 MS patients and 310 NIOND patients) from 16 studies were eligible. A random effects model was used due to significant heterogeneity with *I^2^* = 87%. Pooled results showed an SMD of 1.51 (95% CI: 0.72, 2.30; *p* = 0.0008; Figure 2A). Our results suggest that MS is associated with higher nitrite/nitrate levels within the CSF compared with patients with NIOND. For serum analysis, a total of 185 patients (102 MS patients and 83 NIOND patients) from 5 studies were eligible. Here, a random effects model was used with *I*^2^ = 78%. Pooled results showed an SMD of 0.30 (95% CI: −0.66, 1.26; *p* = 0.4339; Figure 2B). In contrast to the results of the CSF analysis, these findings suggest that nitrite/nitrate levels in the serum of MS and NIOND patients do not differ significantly.

### 3.4. Nitrosative Stress Molecules in Patients with MS and HC

**Figure 3:**
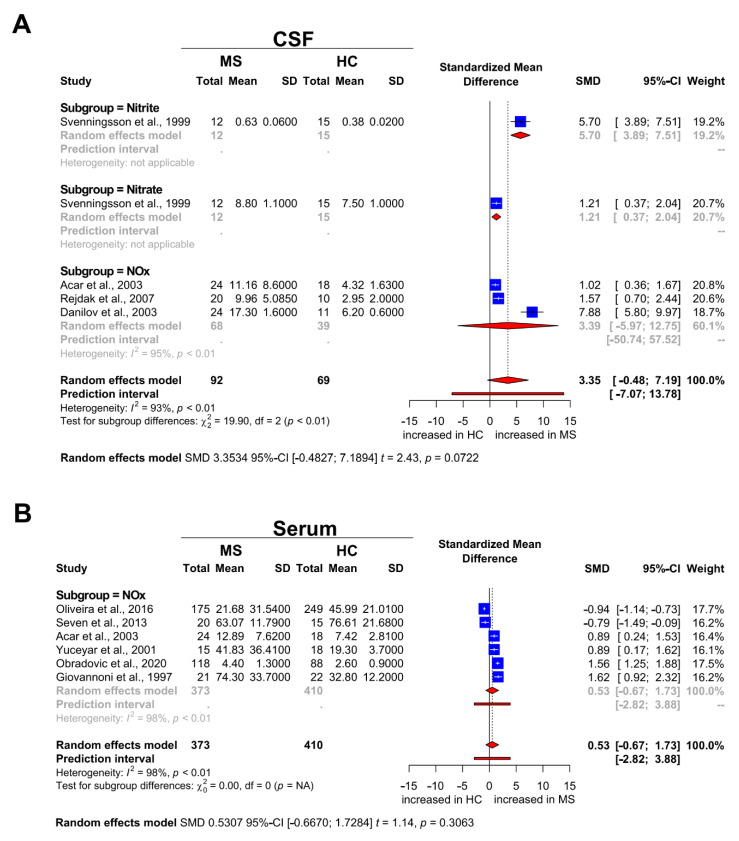
Forest plot displaying the meta-analysis for markers of nitrosative stress in (**A**) the CSF and (**B**) the serum compartment for MS vs. HC, respectively. The dashed vertical line indicates the overall effect, while the solid line indicates the null effect (SMD = 0). Abbreviations: CSF = cerebrospinal fluid, HC = healthy controls, NOx = sum of nitrite and nitrate levels, MS = multiple sclerosis, SD = standard deviation, SMD = standardized mean difference.

For CSF analysis, a total of 134 participants (80 MS patients and 54 HC) from four studies were eligible. The studies showed significant heterogeneity with *I^2^* = 93%, which is why a random effects model was used. There was no statistically significant difference between MS patients and HC, given an SMD of 3.35 (95% CI: −0.48, 7.19; *p* = 0.07; Figure 3A). Our results suggest that the concentration of nitrite/nitrate in the CSF of MS patients is increased compared with HC but does not reach statistical significance. For serum analysis, a total of 783 participants (373 MS patients and 410 HC) from six studies were eligible. Pooled results did not show a statistically significant difference between MS patients and HC with an SMD of 0.53 (95% CI: −0.67, 1.73; *p* = 0.3063; Figure 3B). 

## 4. Discussion

As the diagnosis of MS still requires exclusion of disorders with a similar phenotype, the identification of viable biomarkers to improve diagnostic acuity is of great importance. The clinical differentiation between an MS patient with characteristic symptoms and a healthy asymptomatic individual is easy. The opposite is true for symptomatic patients with MS and NIOND. In the past years, nitrosative stress species have gained increasing scientific attention due to their potential prognostic and diagnostic value. However, the use of nitrosative stress markers in the diagnostic workup for MS is hampered by heterogeneous and, at times, even contradictory results from previous studies [39,40], which is why we conducted this meta-analysis. Comparing MS and NIOND, we observed an overall effect for nitrosative stress species in the CSF but not in the serum. It is tempting to speculate that this could point to a compartment effect of nitrosative stress in the CNS. However, it is known that nitrosative stress molecules, such as nitrite and nitrate, readily cross the blood–brain barrier (BBB) [41]. This apparent contradiction might be explained by the overall scarcity of studies providing data on serum levels of nitrosative stress (see Figure 2B). The comparison between MS and HC followed similar trends as MS and NIOND but did not reach statistical significance probably due to the same problem of underpowering. In general, it is worthwhile to more closely analyze the studies we used for this meta-analysis. Our meta-analysis showed high heterogeneity of the selected studies. This heterogeneity arises from the study population (age, gender, ethnic groups), the sample sizes (varying from 17 to 424), and the application of different MS diagnostic criteria, which evolved during the past 25 years. Of note, some studies did not include data of the mean age and the gender ratio. However, none of the studies performed a subgroup analysis evaluating gender-related differences of the reported results. Another aspect worth discussing are the inclusion criteria for the different groups that were compared. Remarkably, the number of patients with PPMS included was very small, and only a few studies focused exclusively on data from patients with PPMS. Many of the studies included both patients with relapsing and progressive disease courses in their “MS group” or did not clearly differentiate the two MS entities. The same is true for NIOND patients and HC. The inclusion criteria for NIOND and HC groups differ widely from study to study, resulting in cohorts of a very diverse spectrum. Some studies only included tension headache patients in their NIOND group [31], while others included patients with spinal cord tumors or trauma, lumbar disc herniations, epilepsy, and brain tumors [32]. Regarding HC, some studies included CSF or serum samples exclusively from asymptomatic volunteers (see Table 2 and/or Figure 3). Other studies included symptomatic patients complaining of headache or oculomotor palsy with normal CSF [34] findings. For this meta-analysis, we therefore reassigned all studies based on their control group, whereby only asymptomatic individuals were considered as HC and patients with neurological symptoms as NIOND. Beyond the aforementioned aspects, other factors might influence our findings. Both the timepoint of nitrosative stress marker analysis and the presence of an immunomodulatory therapy can influence NOx levels. Accordingly, many of the studies screened determined NOx during relapse and during stable disease and combined the data or did not provide information on the disease stage at all. The same is true regarding treatment. Some studies combine data from therapy-naïve, corticosteroid-treated patients and patients treated with immunomodulatory drugs. Others do not provide any information on treatment. To maintain consistency, we therefore excluded drug intervention studies and preferably included data from MS patients during relapse whenever possible. Furthermore, it should be noted that several different NOx analysis techniques were used, each of them with specific characteristics (see Table 1 and Table 2). Some studies analyzed either nitrate or nitrite. Others provided data on NOx but did not report nitrite and nitrate concentrations separately. Therefore, we decided to report the results of nitrite, nitrate, and NOx separately (see Figure 2 and Figure 3).

In summary, the results of our meta-analysis show that in MS patients, NOx is significantly increased in comparison with patients with NIOND. However, the principal exclusion of NIOND is only the first step on the way to the diagnosis of clinically definite MS. The most challenging next step is to exclude inflammatory other neurological diseases (IOND). This comprises diagnoses such as Behçet’s disease, cerebral vasculitis, acute disseminated encephalomyelitis, brainstem encephalitis, neurosarcoidosis, and connective tissue diseases. Patients with these diseases not only show symptoms and cerebral MRI resembling that of MS patients, but also feature similar CSF findings. Accordingly, the gold standard for an MS biomarker should be its specificity in comparison with IOND, which is mostly missing in the included studies. Future studies will have to address this issue. Translated to clinical practice, our findings argue for including nitrosative stress marker analysis in the CSF workup for MS. In general, CSF analysis has regained the status of a routine procedure since the revision of the McDonald criteria in 2017. In summary, it seems that nitrosative stress molecules in the CSF might be a valuable tool to differentiate MS from NIOND. However, further studies will be needed to corroborate this hypothesis.

## Figures and Tables

**Table 1:** Characteristics of included studies investigating nitrite and/or nitrate levels in multiple sclerosis patients (MS) versus patients with noninflammatory other neurological diseases (NIOND). CSF = cerebrospinal fluid, NOx = sum of nitrite and nitrate, C = concentration in µM, SD = standard deviation, SEM = standard error of mean, IQR = interquartile range, n/a = data not given or not applicable, GC–MS = gas chromatography–mass spectrometry, DAN = diaminonaphthalene, ^a^ = median (range), ^b^ = mean (SEM), ^c^ = median (IQR).

Study	Year	Specimen	MS Patients					NIOND Patients					Assay
			N (m/f)	Mean Age in Yrs (±SD)	C Mean (Nitrite) (±SD)	C Mean (Nitrate) (±SD)	C Mean (NOx) (±SD)	N (m/f)	Mean Age in Yrs (±SD)	C Mean (Nitrite) (±SD)	C Mean (Nitrate) (±SD)	C Mean (NOx) (±SD)	
Cross et al. [19]	1998	CSF	13 (n/a)	n/a	9.98 (34.6)	47.8 (150)	n/a	43 (n/a)	n/a	0.64 (1.21)	3.01 (2.13)	n/a	Fluorometric assay (DAN)
Haghikia et al. [20]	2015	CSF	14 (6/8)	45 (9.6)	2.84 (0.32)	11.3 (0.56)	n/a	26 (12/14)	56.27 (15.8)	2.41 (0.11)	10.5 (0.32)	n/a	GC/MS
Ikeda et al. [21]	1995	CSF	13 (n/a)	34.6 (4.1)	0.62 (0.07)	8.3 (0.6)	n/a	29 (n/a)	54.0 (3.8)	0.63 (0.04)	7.6 (0.7)	n/a	Spectrophotometric assay (Griess)
de Bustos et al. [22]	1999	CSF	11 (5/6)	36.0 (11.3)	n/a	6.2 (2.3)	n/a	25 (10/15)	35.4 (10.7)	n/a	6.9 (3.1)	n/a	Semiautomated assay (Griess)
		serum			n/a	22.7 (6.4)	n/a			n/a	37.1 (23.9)	n/a	
Seven et al. [23]	2013	CSF	20 (7/13)	31.0 (9.6)	n/a	n/a	86.28 (34.1)	15 (6/9)	28.33 (5.31)	n/a	n/a	52.52 (16.5)	Fluorometric assay (Sulphanilamide)
Drulovic et al. [24]	2001	CSF	57 (n/a)	n/a	n/a	n/a	9.5 (1.7)	27 (n/a)	n/a	n/a	n/a	8.7 (2.8)	Spectrophotometric assay (Griess)
Brundin et al. [25]	1999	CSF	9 (n/a)	43.1 (15.0)	9.3 (2.8) ^b^	9.5 (1.7) ^b^	15.1 (3.1) ^b^	8 (2/6)	45.0 (17.0)	2.3 (0.5) ^b^	5.2 (0.5) ^b^	7.5 (0.5) ^b^	Capillary electrophoresis
Johnson et al. [26]	1995	CSF	10 (n/a)	n/a	n/a	n/a	2.59 (0.32) ^b^	10 (n/a)	n/a	n/a	n/a	1.53 (0.22) ^b^	Spectrophotometric assay (Griess)
Yuceyar et al. [27]	2001	CSF	15 (2/13)	29.93 (n/a)	4.85 (3.35)	19.64 (5.59)	24.54 (6.23)	15 (5/10)	43.2 (19.7)	2.61 (1.77)	13.72 (5.17)	16.34 (5.74)	Spectrophotometric assay (Griess)
		serum			5.84 (2.86)	35.98 (35.04)	41.83 (36.41)			2.89 (3.31)	19.75 (6.62)	22.65 (7.8)	
Rejdak et al. [28]	2004	CSF	20 (n/a)	n/a	n/a	n/a	11.3 (4.9)	14 (6/8)	45 (23–74) ^a^	n/a	n/a	6.3 (2.3)	Spectrophotometric assay (Griess)
		serum			n/a	n/a	43.8 (8.3)			n/a	n/a	36.0 (15.9)	
Rejdak et al. [29]	2008	CSF	34 (9/25)	31.0 (20–52) ^a^	n/a	n/a	8.5 (2.5–21.5) ^a^	12 (3/9)	29 (22–50) ^a^	n/a	n/a	2.5 (0.9–7.1) ^a^	Spectrophotometric assay (Griess)
Pirrtilä et al. [30]	2004	CSF	8 (1/7)	28.9 (8.9)	n/a	n/a	1.22 (0.17)	25 (7/18)	47.5 (12.9)	n/a	n/a	1.47 (0.82)	Spectrophotometric assay (Griess)
Danilov et al. [31]	2003	CSF	24 (6/18)	43.5 (19–60) ^a^	7.7 (1.1) ^b^	9.6 (0.7) ^b^	17.3 (1.6) ^b^	8 (2/6)	44.7 (26–66) ^a^	n/a	5.4 (0.3) ^b^	7.4 (0.5) ^b^	Capillary electrophoresis
Miljkovic et al. [32]	2002	CSF	50 (13/37)	35.1 (10.6)	n/a	n/a	9.48 (1.98)	23 (n/a)	n/a	n/a	n/a	8.49 (1.33)	Spectrophotometric assay (Griess)
Giovannoni et al. [33]	1997	serum	21 (5/16)	40.6 (10.7)	n/a	n/a	74.3 (33.7)	14 (7/7)	47.8 (17.8)	n/a	n/a	41.1 (12.3)	Spectrophotometric assay (Griess)
Calabrese et al. [34]	2002	CSF	15 (3/12)	31.0 (7.3)	n/a	n/a	9.4 (1.1) ^b^	15 (2/13)	32.4 (11.0)	n/a	n/a	5.2 (0.9) ^b^	Spectrophotometric assay (Griess)
Sellebjerg et al. [35]	2002	CSF	35 (5/30)	38 (32.0–43.0) ^c^	n/a	n/a	5.5 (3.6–9.1) ^c^	15 (4/11)	45 (45.0–60.0) ^c^	n/a	n/a	3.3 (1.4–4.9) ^c^	Spectrophotometric assay (Griess)
		serum			n/a	n/a	34.3 (27.0–47.3) ^c^			n/a	n/a	40.7 (36.5–51.1) ^c^	

**Table 2:** Characteristics of included studies investigating nitrite and/or nitrate levels in multiple sclerosis patients (MS) versus healthy controls (HC). CSF = cerebrospinal fluid, NOx = sum of nitrite and nitrate levels, C = concentration in µM, SD = standard deviation, SEM = standard error of mean, IQR = interquartile range, n/a = data not given or not applicable, GC–MS = gas chromatography–mass spectrometry, ^a^ = median (range), ^b^ = mean (SEM).

Study	Year	Specimen	MS Patients					HC					Assay
			N (m/f)	Mean Age in Yrs (±SD)	C Mean (Nitrite) (±SD)	C Mean (Nitrate) (±SD)	C Mean (NOx) (±SD)	N (m/f)	Mean Age in Yrs (±SD)	C Mean (Nitrite) (±SD)	C Mean (Nitrate) (±SD)	C Mean (NOx) (±SD)	
Svenningsson et al. [36]	1999	CSF	12 (n/a)	n/a	0.63 (0.06) ^b^	8.8 (1.1) ^b^	n/a	15 (n/a)	n/a	0.38 (0.02) ^b^	7.5 (1.0) ^b^	n/a	GC–MS
Rejdak et al. [37]	2007	CSF	20 (6/14)	28 (21–46) ^a^	n/a	n/a	9.1 (2.5–21.5) ^a^	10 (4/6)	29 (20–40) ^a^	n/a	n/a	2.2 (0.9–7.1) ^a^	Spectrophotometric assay (Griess)
Acar et al. [38]	2003	CSF	24 (9/15)	30.2 (8.3)	n/a	n/a	11.16 (8.6)	18 (8/10)	32.0 (2.34)	n/a	n/a	4.32 (1.63)	Spectrophotometric assay (Griess)
		serum			n/a	n/a	12.89 (7.62)			n/a	n/a	7.42 (2.81)	
Danilov et al. [31]	2003	CSF	24 (6/18)	43.5 (19–60) ^a^	7.7 (1.1) ^b^	9.6 (0.7) ^b^	17.3 (1.6) ^b^	11 (3/8)	40.0 (27–58) ^a^	1.9 (0.4) ^b^	4.4 (0.3) ^b^	6.2 (0.6) ^b^	Capillary electrophoresis
Oliveira et al. [39]	2017	serum	175 (n/a)	n/a	n/a	n/a	21.68 (31.54)	249 (72/177)	36.7 (10.9)	n/a	n/a	45.99 (26.01)	Spectrophotometric assay (Griess)
Seven et al. [23]	2013	serum	20 (7/13)	31.0 (9.6)	n/a	n/a	86.28 (34.1)	15 (5/10)	30.2 (5.51)	n/a	n/a	76.61 (21.68)	Fluorometric assay (Sulphanilamide)
Yuceyar et al. [27]	2001	serum	15 (2/13)	29.93 (n/a)	5.84 (2.86)	35.98 (35.04)	41.83 (36.41)	18 (8/10)	33.12 (5.5)	2.1 (3.3)	17.2 (4.1)	19.3 (3.7)	Spectrophotometric assay (Griess)
Obradovic et al. [40]	2020	serum	59 (24/35)	40.0 (10.2)	n/a	n/a	4.5 (1.5)	88 (36/52)	38.9 (9.0)	n/a	n/a	2.6 (0.9)	Semiautomated assay (Griess)
Giovannoni et al. [33]	1997	serum	21 (5/16)	40.6 (10.7)	n/a	n/a	74.3 (33.7)	22 (11/11)	33.8 (7.4)	n/a	n/a	32.8 (12.2)	Spectrophotometric assay (Griess)

## Data Availability

Data will be provided on request.
